# Glycemic Changes Related to Arsenic Exposure: An Overview of Animal and Human Studies

**DOI:** 10.3390/nu16050665

**Published:** 2024-02-27

**Authors:** Geovanna Beatriz Oliveira Rosendo, Rannapaula Lawrynhuk Urbano Ferreira, Séphora Louyse Silva Aquino, Fernando Barbosa, Lucia Fatima Campos Pedrosa

**Affiliations:** 1 Postgraduate Program in Health Sciences, Federal University of Rio Grande do Norte, Natal 59012-570, RN, Brazil; geovanna.rosendo.115@ufrn.edu.br (G.B.O.R.); rannapaula.lawrynhuk.107@ufrn.edu.br (R.L.U.F.); sephora.aquino.087@ufrn.edu.br (S.L.S.A.); 2 Department of Clinical Analyses, Toxicology and Food Sciences, School of Pharmaceutical Sciences of Ribeirão Preto, University of São Paulo, Ribeirão Preto 14040-903, SP, Brazil; fbarbosa@fcfrp.usp.br; 3 Department of Nutrition, Federal University of Rio Grande do Norte, Natal 59078-970, RN, Brazil

**Keywords:** arsenic exposure, diabetes mellitus, hyperglycemia, glucose intolerance

## Abstract

Background: Arsenic (As) is a risk factor associated with glycemic alterations. However, the mechanisms of action and metabolic aspects associated with changes in glycemic profiles have not yet been completely elucidated. Therefore, in this review, we aimed to investigate the metabolic aspects of As and its mechanism of action associated with glycemic changes. Methods: We searched the PubMed (MEDLINE) and Google Scholar databases for relevant articles published in English. A combination of free text and medical subject heading keywords and search terms was used to construct search equations. The search yielded 466 articles; however, only 50 were included in the review. Results: We observed that the relationship between As exposure and glycemic alterations in humans may be associated with sex, smoking status, body mass index, age, occupation, and genetic factors. The main mechanisms of action associated with changes induced by exposure to As in the glycemic profile identified in animals are increased oxidative stress, reduced expression of glucose transporter type 4, induction of inflammatory factor expression and dysfunction of pancreatic β cells. Conclusions: Therefore, As exposure may be associated with glycemic alterations according to inter-individual differences.

## 1. Introduction

Endocrine disorders are among the harmful effects of heavy metal exposure on human health, resulting in changes in the hypothalamus-pituitary axis and the interruption of the secretion of hormones such as thyroid-stimulating, luteinizing, and adrenocorticotropic hormones, and prolactin [1]. Exposure to endocrine-disrupting chemicals, including arsenic (As), is associated with diabetes mellitus (DM) etiology. As it accumulates in the liver, kidney, and pancreas, it has harmful effects on carbohydrate metabolism pathways, especially glycolysis, glycogenesis, and gluconeogenesis [2].

Environmental exposure to As has been observed in several countries, including the United States (USA) [3], Croatia [4], Mexico [5], and Spain [6]. As contamination in Brazil has been recorded in soils, sediments, and water sources in the northern, southern, and southeastern regions, mainly arising from anthropogenic actions [7].

As is highly toxic and can be found in nature in the elemental forms As0, As3+, and As5+ and as organic arsenobetaine, arsenosugars, and arsenolipids, predominantly in seafood [8]. The inorganic forms (iAs), arsenite and arsenate, are present in drinking water, food, dust, and ambient air [9]. Considerable iAs concentrations have been detected in different types of rice depending on the cultivation method, processing, and country of production [10].

Organic forms of As are nontoxic because they are not metabolized and are rapidly excreted [11]. In contrast, iAs undergoes several methylations and are converted into monomethyl arsenic (MAsIII and MAsV) and dimethyl arsenic (DMAsIII and DMAsV) compounds [12], which are excreted in the urine together with unchanged As [13].

Studies in animal models exposed to iAs have identified increased fasting glucose and insulin levels [12], glucose intolerance, reduced insulin resistance index (HOMA-IR) [13], and oxidative damage [14]. Yang et al. [15] identified low to moderate concentrations of As that were not associated with the development of type 2 DM (T2DM) throughout life. Another study observed that the total urinary concentration of As was positively correlated with the prevalence of T2DM and prediabetes [16].

However, the mechanisms of action associated with these effects have not yet been elucidated. Therefore, in this review, we aimed to investigate the metabolic aspects of As and its relationship with glycemic changes.

## 2. Materials and Methods

The PubMed (MEDLINE) database was used as the primary source of potentially relevant studies. Google Scholar was used as the secondary source, with searches limited to 100 reports, sorted by relevance ranking [17]. We searched databases for articles published between January 1998 and February 2023. The eligibility criteria included full-text articles on animal and human studies published in English. Narrative reviews, systematic reviews, commentaries, correspondences, editorials, in vitro studies, and studies with self-reported DM diagnoses were excluded.

A search was conducted using a combination of free text and medical subject heading (MeSH) search terms and keywords, namely “Diabetes mellitus”, “Prediabetic states”, “Hyperglycemia”, “Glucose intolerance”, “Arsenic”, and “Arsenite”, based on each database characteristic. Both the keywords and search terms were used to construct the search equations. The reports were transferred to the Rayyan-Intelligent Systematic Review application developed by the Qatar Computing Research Institute [18] for the selection procedures. Two investigators independently selected the studies by analyzing the titles, abstracts, and keywords.

## 3. Results

**Figure 1:**
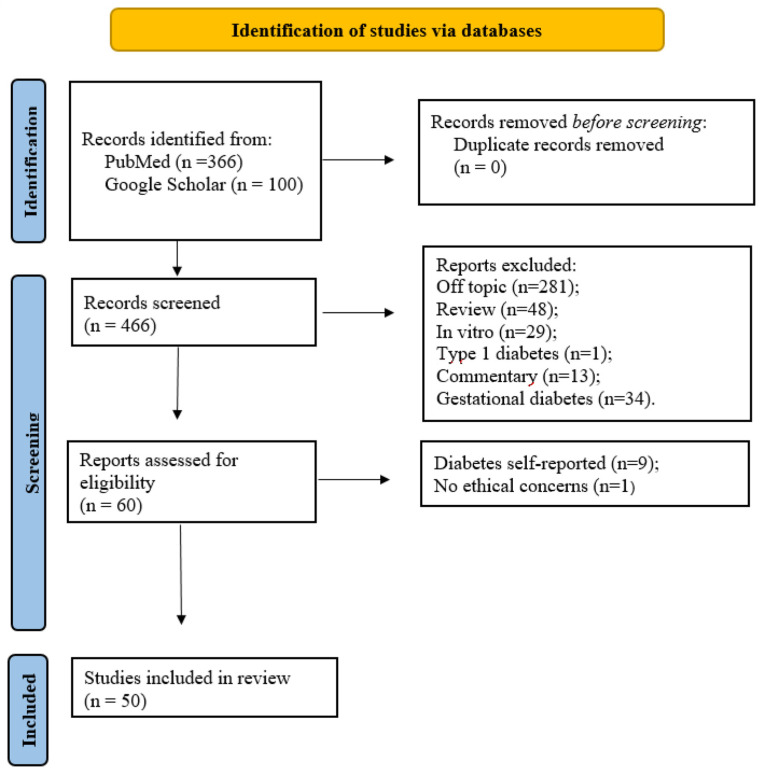
Study selection flowchart. Adapted from: Page MJ, McKenzie JE, Bossuyt PM, Boutron I, Hoffmann TC, Mulrow CD, et al. The PRISMA 2020 statement: an updated guideline for reporting systematic reviews. BMJ 2021;372: n71. doi: 10.1136/bmj.n71.

The search yielded 466 articles; however, only 50 were included in this narrative review (Figure 1).

A total of 16 studies conducted on animals used mice [13,14,19,20,21,22,23,24,25,26], Wistar rats [12,27,28,29], Sprague-Dawley rats [30], and a diversity of outbred male mice [31]. The animals were exposed to different As doses ranging from 0.20 to 800 mg/L for different periods, with the lowest exposure being 15 min and the highest being 12 months, and these studies were published between 2006 and 2022 (Table 1).

The 34 studies performed on humans were cross-sectional [6,10,16,32,33,34,35,36,37,38,39,40,41,42,43,44,45], cohort [5,6,15,46,47], or case-control [4,48,49,50,51,52,53,54,55,56,57]. They were published between 2007 and 2022 in the following countries: USA (n = 12), Mexico (n = 6), China (n = 3), Bangladesh (n = 3), Pakistan (n = 2), Korea (n = 2), Croatia (n = 1), Canada (n = 1), Spain (n = 1), Iran (n = 1), Serbia (n = 1), and Cambodia (n = 1). Among the included studies, only three [15,20,43] did not identify an association between As exposure and glycemic alterations (Table 2).

**Table 2:** Characteristics of the human studies included in the review.

Author/Year	Region	Study Design	Sample Size	Age (Years)	Main Results
Coronado-González et al., 2007 [48]	Mexico	Case-control	Men and women (n = 400)	≥30	Dose-response relationship between As concentrations in urine and T2DM.
Navas-Acien et al., 2008 [35]	USA	Cross-sectional	Men and women (n = 788)	≥20	Association between exposure to As and the prevalence of T2DM.
Kim and Lee 2011 [46]	Korea	Cohort	Men and women (n = 1677)	≥20	Urinary associations increased the risk of DM, mainly in females.
Gribble et al., 2012 [3]	USA	Cohort	Men and women (n = 3925)	45–74	As was positively associated with hemoglobin A1c concentrations in participants with DM.
Rhee et al., 2013 [10]	Korea	Cross-sectional	Men and women (n = 3602)	≥20	Significantly higher total urinary As concentration in females, the elderly, and residents of urban areas.
Drobná, Del Razo, and García–Vargas 2013 [33]	Mexico	Cross-sectional	Men and women (n = 255)	≥5	Individuals with the AS3MT/M287T and G4965C variants had higher concentrations of DMAIII.
Kim, Mason, and Nelson 2013 [38]	USA	Cross-sectional	Men and women (n = 300)	≥25	Fasting plasma glucose was negatively correlated with % MMA and positively correlated with total As.
Pan et al., 2013 [52]	Bangladesh	Case-control	Men and women (n = 919)	DM: 40.0 (14.0) C: 33.0 (18.0)	Genetic susceptibility to T2DM likely induced by As.
Pan et al., 2013 [53]	Bangladesh	Case-control	Men and women (n = 933)	DM: 33.0 (18.0) C: 40.0 (13.5)	Synergistic effect between As exposure, smoking, and BMI resulted in the highest risk of T2DM.
Bailey et al., 2013 [32]	Mexico	Cross-sectional	Women (n = 16)	*	Methylation patterns of DM-related genes were associated with urinary concentrations of iAs metabolites.
Jovanovic et al., 2013 [37]	Serbia	Cross-sectional	Population of Middle Banat region, Serbia (*)	Men: Exposed: 60.1 (10.9) Not exposed: 60.8 (11.2) Women: Exposed: 61.7 (9.8) Not exposed: 63.5 (10.7)	Higher incidence rates of T2DM in the population exposed to As.
Díaz-Villaseñor et al., 2013 [50]	Mexico	Case-control	Men and women (n = 72)	35–65	Chronic exposure to iAs reduced β cell function.
Huang et al., 2014 [36]	Cambodia	Cross-sectional	Men and women (n = 142)	40.4	Water intake with As concentrations above the median (907.25 μg/L) was associated with an increased risk of DM.
Peng, Harlow, and Park 2015 [43]	USA	Cross-sectional	Men and women (n = 835)	12–19	No associations between HOMA-IR and As, iAs, or DMA.
Martin, González-Horta, and Rager 2015 [5]	Mexico	Cohort	Men and women (n = 1165)	≥18	Difference in the metabolites found in the urine of individuals with or without DM.
Feseke et al., 2015 [16]	Canada	Cross-sectional	Men and women (n = 3151)	20–79	Urinary As concentration was positively associated with the prevalence of T2DM and prediabetes.
Park et al., 2016 [41]	USA	Cross-sectional	Men and women (n = 221)	52.5	Total urine was associated with high concentrations of fasting blood glucose.
Grau-Perez et al., 2017 [58]	USA	Cohort	Men and women (n = 1838)	24–47	Interaction of one-carbon metabolism nutrients and % MMA with an AS3MT genetic variant.
Grau-Perez, Navas-Acien, and Galan-Chilet 2018 [6]	Spain	Cross-sectional	Men and women (n = 1451)	≥20	Positive association between total As in urine and the prevalence of DM.
Spratlen et al., 2018 [47]	USA	Cohort	Men and women (1458)	>14	Participants who developed DM were older, had higher % DMA, BMI, HOMA-IR, and waist circumference and lower % MMA.
Yang et al., 2019 [15]	USA	Cohort	Men and women n = (4102)	20–32	Low to moderate concentrations of As in the nails were not associated with the risk of developing DM.
Spratlen et al., 2019 [44]	USA	Cross-sectional	Men and women (n = 935)	14–23	Association of lower % MMA and higher % DMA with DM-related outcomes may be influenced by carbon metabolism status.
Paul et al., 2019 [42]	Bangladesh	Cross-sectional	Men and women (n = 641)	18–60	Dose-dependent association between As exposure and hyperglycemia, especially in females.
Rehman, Fatima, and Akash 2019 [55]	Pakistan	Case-control	Men and women (n = 150)	≥18	As was positively associated with increased risk of DM when adjusted for sex, age ≥ 60 years, education, and smoking.
Zhang et al., 2020 [57]	China	Case-control	Men and women (n = 1248)	≥18	Patients with higher urinary % As were more likely to have DM.
Lucio, Barbir, and Vučić Lovrenčić 2020 [4]	Croatia	Case-control	Men and women (n = 201)	East—C: 49 (14); PD: 64 (7); DM: 64 (10) West—C: 45 (11); PD: 57 (6); DM: 57 (7)	Total As metabolites in urine were positively correlated with hemoglobin A1c.
Idrees and Batool 2020 [51]	Pakistan	Case-control	Men and women (n = 200)	26–80	Association between As exposure and T2DM development.
Wu et al., 2021 [56]	USA	Case-control	Men and women (n = 190)	56 (51–64)	Increase in % MMA was positively associated with prediabetes and DM.
Arab, Arbabi, and Ziarati 2021 [48]	Iran	Case-control	Men and women (n = 200)	>40	Urinary As concentration was four times higher in patients with T2DM.
Li, Wang, and Park 2021 [39]	USA	Cross-sectional	Men and women (n = 5469)	≥20	Rice consumption was positively associated with higher urinary DMA concentration but inversely associated with MMA.
Liu et al., 2022 [40]	China	Cross-sectional	Men and women (n = 436)	>18	As exposure had a disruptive effect on glucose homeostasis and resulted in an elevated inflammatory response.
Rangel-Moreno et al., 2022 [54]	Mexico	Case-control	Women (n = 681)	36–88	T2DM prevalence was associated with iAs metabolism but not with urinary As concentration.
Fan et al., 2022 [34]	China	Cross-sectional	Men and women (n = 938)	>20	Age ≥ 60 years, the female gender, and high level of urinary iAs were correlated with a risk of T2DM, whereas the A allele and AA genotype of the KEAP1 SNP rs11545829 may be a protective factor.
Zhou, Zhao, and Huang 2022 [45]	USA	Cross-sectional	Men and women (n = 815)	20–79	Total As exposure was positively correlated with insulin resistance.

DM: Diabetes mellitus; C: Control; DM2: Diabetes mellitus type 2; PD: Prediabetes; BMI: Body mass index As: Arsenic; iAs: inorganic arsenic; HOMA-IR: Insulin resistance index; hemoglobin A1c: Glycosylated hemoglobin; MMA: Monomethylarsonic acid; DMA: Dimethylarsinic acid; AS3MT: Arsenic (+3 oxidation state) methyltransferase. * Information was not reported in the study.

We noted that the relationship between exposure to As and glycemic changes in humans might be associated with place of residence [10], gender [10,34,42,46], smoking [51], body mass index (BMI) [34,52], age [34,47], educational level [55], occupation [10], and genetic factors [5,6,32,33,34,52]. The possible associated mechanisms with glycemic responses are related to mitochondrial dysfunction [12], reduced expression of glucose transporter type 4 (GLUT4) [14,25], induction of inflammatory factors [28], reduction in gamma-type peroxisome gene expression (Pparγ) [21], increase in oxidative stress and, consequently, in antioxidant enzymes [19,27,28,29], dysfunction of pancreatic β cells [50], increased gluconeogenesis [14], and changes in the gut metabolome and microbiome [22].

## 4. Discussion

### 4.1. Forms and Sources of As

As is a toxic metalloid from natural and anthropogenic sources and is found in water, soil, and air [57]. In nature, As is present in the elemental form As0, or in combination with other metalloids, in the trivalent (As3+), pentavalent (As5+), and organic forms [11] iAs, such as arsenite and arsenate, are found in drinking water, food, dust, and ambient air [9]. In contrast, organic forms such as arsenobetaine, arsenosugars, and arsenolipids are found mainly in seafood [35].

Industrial pollution [59], mineral extraction, and use of pig feed additives [10], fertilizers, and pesticides [60] are environmental sources of As contamination. A study carried out in the Middle Banat (Serbia) region identified a median As concentration of 56.1 μg/L in the water of public supply systems [37].

Based on the total dietary exposure to iAs, the maximum tolerated intake for humans is estimated to be between 2 and 7 g/kg body weight/day [61]. Drinking water is a significant contributor to the dietary exposure to total iAs. Among foods with the highest As contamination, rice ranks at the top [39] and, when adulterated, milk can contain water containing As [62].

Depending on the concentration, As contamination can also occur through food preparation and crop irrigation. The urinary concentration of As may vary according to demographic characteristics and lifestyle, being higher in agricultural workers, forestry workers, fishers, artisans, operators of installations and machines, and assemblers [10]. Regarding lifestyle, lower concentrations of urinary As have been detected among current smokers and non-drinkers [10].

### 4.2. As Absorption, Metabolism, and Excretion

**Figure 2:**
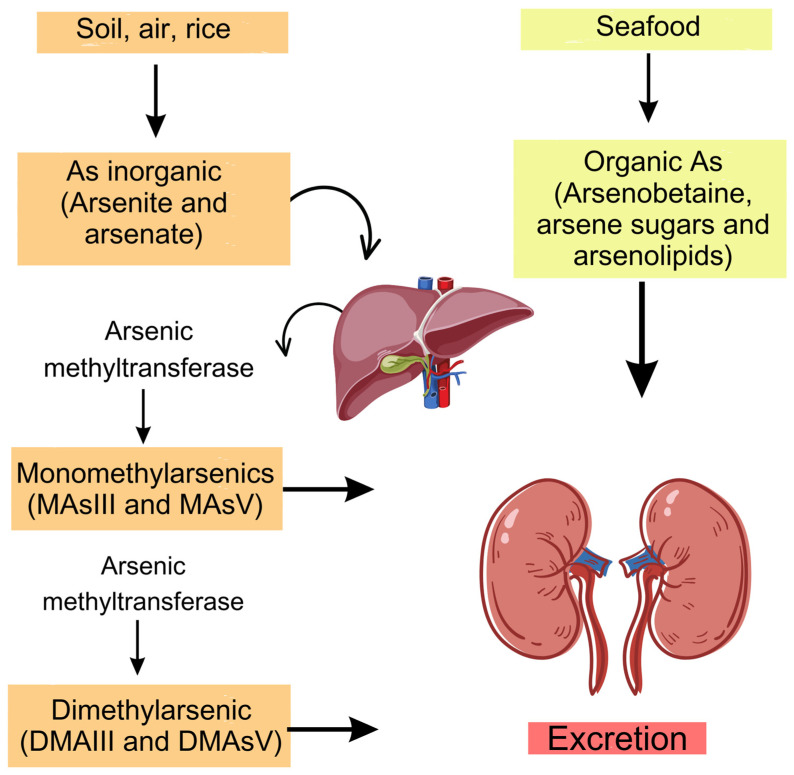
Metabolism of different forms of As. The organic form is excreted and unchanged, whereas iAs undergoes methylation and reduction processes by the enzyme arsenic methyltransferase (AS3MT). Created with https://www.Canva.com. (Figure illustration by Rosendo, G.B.R).

The different forms of As are metabolized in different ways: organic As is excreted unchanged through the urine [35], whereas iAs is methylated by the enzyme arsenic methyltransferase (AS3MT) [9]. Initially, iAs is methylated and reduced to monomethyl arsenic (MAsIII and MAsV), and the process is repeated, forming dimethyl arsenic (DMAsIII and DMAsV) [35] (Figure 2).

Thus, As metabolism is directly associated with methylation reactions and, as a consequence, requires the generation of methyl groups, the availability of which depends on essential nutrients such as folate, vitamin B12, vitamin B6, vitamin B2, and methionine [6,44].

iAs poses a greater risk to human health, whereas organic As forms are considered nontoxic because they are rapidly excreted [10]. Furthermore, other variables, such as valence, physical state, solubility, purity, absorption, and elimination rate, also influence As toxicity [63].

Inter-individual differences in the As methylation capacity have nongenetic determinants, including sex, smoking, diet, and BMI [64]. Women have a higher % DMA than men, never-smokers generally have a higher % DMA than current smokers, dietary deficiencies of folate and vitamins are associated with lower % DMA, and obese individuals have a higher % DMA [9].

Regarding nutritional status and BMI, a lower intake of methyl-group-containing diet may result in lower As methylation [65]. Individuals with higher BMI have been found to consume more cofactors used in As metabolism [66], and that body fat may interfere with As storage [64].

Hormonal differences, mainly in estrogen levels, likely explain the sex differences in As methylation capacity [64]. With regard to smoking, the chemical substances in cigarettes compete for some enzymes or cofactors involved in As methylation processes [64]. Furthermore, aging may be related to alterations in As metabolism owing to functional disturbances in metabolite excretion [64]. Genetic factors are also involved in As metabolism, and higher urinary concentrations of DMAs have been identified in individuals harboring the M287T and G4965C variants of AS3MT [32].

As metabolism is speculated to differ under DM conditions. Diabetic mice (C57BKS/Lepr db) exposed to higher concentrations of iAs exhibited lower urinary excretion and a higher degree of As methylation than healthy mice [14]. From this perspective, genetic variations could increase susceptibility to DM2 among individuals exposed to iAs [51].

### 4.3. Exposure to As and Glycemic Alterations

Epidemiological studies have reported that exposure to As may be associated with increased HOMA-IR index [47], hemoglobin A1c [3,4], fasting glucose level [12,31,41], insulin resistance [21,31,36,45], and glucose intolerance [13,21,23,30]. In contrast, mice (C57BL/6) exposed to iAs in drinking water (5 and 50 ppm) for 18 weeks showed no significant changes in serum glucose and insulin concentrations. These results were discussed in terms of methodological aspects, such as the lack of measurement of glucose tolerance and euglycemic-hyperinsulinemic clamping [20].

Peng et al. (2015) [43] found no changes in HOMA-IR in adolescents exposed to low As levels [43]. Accordingly, in a population with low to moderate As exposure, As concentrations in the toenails were not associated with fasting blood glucose and insulin levels and HOMA-IR [15]. Individuals from Bangladesh exposed to moderate and high doses of As were found to be more vulnerable to hyperglycemia [42].

In animal studies, acute exposure of Sprague-Dawley rats to 2, 4, and 8 mg/kg As for 15 to 120 min increased their blood glucose concentrations [30]. Gong et al. (2019) [23] found that exposure to 0.25 ppm iAs caused glucose intolerance in C57BL/6 mice, whereas the group exposed to 2.5 ppm iAs did not show significant changes in glucose tolerance [23]. The inconsistency between these findings implies a dose-time-response relationship between As exposure and glycemic alterations.

Interventions using antioxidant compounds can prevent the harmful effects of As toxicity. Rezaei et al. (2017) [30] found that pretreatment of Sprague-Dawley rats with 40 and 80 mg/kg N-acetylcysteine prevented As-induced glucose disturbances.

### 4.4. Mechanism of Action for As-Induced Glycemic Changes

#### 4.4.1. Mitochondrial Dysfunction and Expression of Pro-Inflammatory Factors

The toxicity of As is related to its chemical form, oxidation state, and exposure dose [67]. The biotransformation process of As involves methylation, which results in more toxic final metabolites. The increased acute toxicity of methylated trivalent As intermediates suggests that As methylation is not simply a detoxification mechanism [67].

Reactive Oxygen Species (ROS) mediated oxidative damage is a common denominator in As pathogenesis [68].As-triggered T2DM has been reported to contribute to the mitochondrial overproduction of ROS [12], and compensatory As-induced oxidative stress leads to an increase in the activity of antioxidant enzymes, such as total glutathione [27,29], superoxide dismutase, catalase, and glutathione-S-transferase activity [27]. As a result of oxidative stress, tissue injury may occur, causing an increase in the inflammatory focus and release of tumor necrosis factor alpha (TNF-α) [28].

ROS are involved in intracellular signaling processes, regulation of cellular activity, and immune responses [69]. Increased ROS stimulate inflammatory responses that damage key cellular components, including lipids, proteins, and deoxyribonucleic acid (DNA) [69,70].

With increased inflammation, TNF-α may play a role in causing fatty insulin resistance in patients with T2DM [71]. Exposure to As results in increased oxidative stress, and consequently, apoptosis of human hepatocytes of the Chang lineage [72].

Additionally, a study conducted in Wistar rats showed that exposure to As_2_O_3_ counteractingly increased the concentration of sirtuin 3, which is responsible for safeguarding the mitochondria against damage induced by free radicals. This illustrates the diabetogenic potential of As, as it disrupts mitochondrial respiration by reducing membrane potential, and consequently, cellular respiration and signaling [12,70].

#### 4.4.2. Damage Caused to DNA

The potential genotoxic damage related to iAs exposure has also been studied. iAs may be associated with single-strand DNA breaks, the formation of apurinic and apyrimidine sites, oxidation of DNA bases, DNA-protein cross-linking, and chromosomal aberrations [68]. Industrial workers exposed to As face a significant risk of genetic instability due to damage caused by oxidative stress, which is induced by the downregulation of the OGG1 and HPRT genes [73].

Furthermore, epigenetic changes have been suggested to play a significant role in the mechanism of action of iAs by altering methylation patterns [74]. A relationship has been identified between exposure to low and moderate concentrations of As and the methylation of SLC7A11, a gene associated with the biosynthesis of glutathione, a crucial endogenous antioxidant that may provide protection against As-induced oxidative stress [74].

However, further studies are required to determine whether these DNA methylation profiles provide mechanistic insights into the development of iAs-associated diseases or serve as biomarkers for iAs exposure in humans [32].

#### 4.4.3. Reduced GLUT4 Expression and Reduced PPARγ Expression

Li et al. (2021) investigated the response of adipose cells to exposure to iAs and MAs and observed the suppression of PKB/Akt phosphorylation and interference with GLUT4 translocation. Therefore, when GLUT4 recruitment to the membrane becomes unviable, insulin-stimulated glucose uptake is compromised [25].

Prolonged exposure to iAs results in glucose intolerance, insulin resistance and lower PPARγ expression in mice. Impaired expression of PPARγ results in repression of adipocyte differentiation, increased lipolysis, and decreased insulin sensitivity [63].

#### 4.4.4. Increased Gluconeogenesis and Pancreatic β-Cell Dysfunction

In individuals with T2DM, an inverse association was identified between urinary As concentration and the function of β-cells, which are possibly more susceptible to damage caused by iAs exposure than those in healthy individuals [50]. It is noteworthy that pancreatic β cells are highly sensitive to oxidative stress, resulting in the induction of chronic inflammation and cell apoptosis [57]. Experimental studies have demonstrated that As induces β-cell destruction, thereby impairing insulin production and release and glucose-driven insulin secretion [14].

Furthermore, As exposure is associated with increased gluconeogenesis, which may contribute to increased fasting blood glucose levels and lower glucose tolerance [14]. The detrimental effects associated with carbohydrate metabolism pathways, such as glycolysis, glycogenesis, and gluconeogenesis, may occur due to the tendency of As to primarily accumulate in the liver, kidneys, and pancreas [75,76].

This accumulation induces alterations in enzymatic configuration, resulting in the modification of the active site and, consequently, enzyme activity [75,76]. Notably, As has the capacity to alter the enzymatic activity of pyruvate dehydrogenase, thereby interfering with the Krebs cycle and inhibiting oxidative phosphorylation, ultimately resulting in cellular damage [76].

#### 4.4.5. Changes in the Metabolome and Intestinal Microbiome

Li et al. (2019) identified that exposure to heavy metals, including As, is associated with significant global alterations in the intestinal microbiome, affecting bacterial genera associated with T2DM [22]. Exposure to heavy metals slows growth and modifies the structure of phyla within the intestinal microbiome, affecting the biological functions of the microbiota, including metabolism and immunity [77]. Immunomediated reactions triggered by changes in the microbiota composition are likely to facilitate the development of DM in predisposed individuals [78].

In a recent Strong Heart Family Study involving 59 participants, it was hypothesized that the one-carbon metabolic (OCM) pathway could influence the relationship between As metabolism and diabetes. After metabolomic analyses, eight metabolites of interest correlated with DM-related outcomes, including LPS 18:2, which is strongly associated with As metabolism and central obesity [44]. Even with promising discoveries, there is still a lack of robust evidence on the subject that elucidates the mechanisms that interconnect exposure to As with changes in the microbiota, metabolomics, and metabolism in DM.

Finally, owing to the cross-sectional design characteristics of most of the articles included in this review, the causal relationship between As exposure and glycemic alterations is unclear. In addition, exposure to As was mostly based on the quantification of total As in a single urine and/or blood sample. Therefore, future studies with a longitudinal design are suggested to quantify the concentrations of not only total As but also of other forms of As at different time points.

## 5. Conclusions

Exposure to As may be associated with glycemic alterations, such as hyperglycemia and insulin resistance, in animals and humans. In addition, there is an increase in hemoglobin A1c level and the risk of DM and prediabetes in humans, according to inter-individual factors. The main mechanisms of action associated with glycemic profiles identified in animals change due to As exposure are increased oxidative stress, reduced GLUT4 expression, induction of expression of inflammatory factors, and pancreatic β cell dysfunction. However, more studies are needed to elucidate the relationship between the dose and duration of exposure to As for outcomes related to changes in the glycemic profile. Of note, an animal model study has shed light on the role of antioxidants in preventing glycemic changes associated with As exposure.

## Figures and Tables

**Table 1:** Characteristics of animal studies included in the review.

Author/Year	Experimental Model	Treatment/Duration	Main Results
Izquierdo-Vega et al., 2006 [29]	Male Wistar rats	Water or sodium arsenite; 1.7 mg/kg. 12 h. 90 days	Hyperglycemia, hyperinsulinemia, and low insulin sensitivity. Increased total glutathione and lipoperoxidation in the pancreas of the group exposed to iAs
Patel and Kalia 2013 [27]	Albino Wistar Rats	Distilled water, 1.5 mg/kg^−1^ b. wt or 5.0 mg/kg^−1^ b. wt of sodium arsenite. 4 weeks	Increased superoxide dismutase (SOD), catalase, and glutathione-S-transferase activity
Liu et al., 2014 [14]	Healthy (C57BLKS/J) and diabetic (C57BKS/Lepr^db^) mice	Deionized water or 3 mg/L iAs. 16 weeks	iAs increased oxidative stress and inflammation in liver and pancreas of healthy mice. It also increased gluconeogenesis and reduced gene expression of GLUT4.
Rezaei et al., 2017 [30]	Male Sprague-Dawley rats	Control, As, As + N-acetylcysteine, carvedilol, carvedilol + As, propranolol, or propranolol + As. Acute exposure: 2, 4, or 8 mg/L of As for 15 to 120 min. Chronic exposure: 0.20, 40, or 60 ppm for 8 weeks (0.20 mg/L, 40 mg/L, 60 mg/L) or 200, 400, or 800 ppm for 20 weeks (200 mg/L, 400 mg/L, 800 mg/L)	Acute exposure to As-induced glucose intolerance. Preventive role of N-acetylcysteine against glycemic changes caused by As.
Yin et al., 2017 [19]	Diabetic and healthy mice (C57BLKS/J), age—7 weeks	Deionized water or sodium arsenite 3 mg/L. 16 weeks	Increased glutathione peroxidase concentration in diabetic mice exposed to iAs.
Song et al., 2017 [20]	Healthy mice (C57BL/6), age—4 weeks	Water, water + 5 ppm (5 mg/L) of iAs, or water + 50 ppm (50 mg/L). 18 weeks	No changes in serum insulin and glucose concentrations. Adiponectin reduction.
Souza et al., 2018 [28]	Healthy and diabetic male Wistar rats, age—70 days	Diabetes was induced using streptozotocin. Exposed to saline solution (0.9% NaCl) or 10 mg/L of sodium arsenate. 40 days	iAs exposure increased SOD and glutathione s-transferase activity in healthy and diabetic rats. iAs caused a hepatic inflammatory reaction with increased TNF-α.
Kirkley et al., 2018 [13]	Male mice (C57BL/6J), age—7 to 8 weeks	Water or water + 50 mg L of sodium arsenite. 8 weeks	Mice exposed to As exhibited glucose intolerance without altering overall insulin sensitivity. 28% reduction in HOMA-IR.
Zuo et al., 2019 [21]	Female mice (C57BL/6), 10 weeks	Mice exposed to 0, 5, or 20 ppm (5 or 20 mg/L) iAs in drinking water. 17 weeks	Prolonged exposure to iAs caused glucose intolerance, insulin resistance, and lower PPARγ.
Li et al., 2019 [22]	Mice (C57BL/6), 5 weeks	Water, water + 50 ppm cadmium chloride, or water + 50 ppm (50 mg/L) sodium arsenite. 2 weeks	Exposure to iAs caused overall changes in the intestinal metabolome and microbiome.
Gong et al., 2019 [23]	Mice (C57BL/6), 8 weeks	Deionized water + 0.25 ppm (0.25 mg/L) sodium arsenite or deionized water + 2.5 ppm (2.5 mg/L) sodium arsenite. 15 weeks	Exposure to 0.25 ppm iAs caused glucose intolerance. Exposure to 2.5 ppm iAs not significant for glucose tolerance.
Rezaei et al., 2019 [12]	Male Wistar rats, 10 weeks	Normal diet, diet + As trioxide (7 mg/kg), varying with or without the presence of metformin or berberine. Every 2 days for 8 days	iAs increased fasting glucose and insulin compared to the control group. Increased SIRT3 concentration and mitochondrial dysfunction due to exposure to iAs.
Castriota et al., 2020 [24]	Mice (C57BL/6J), 5 weeks	Drinking water + 300 μg/L (0.3 mg/L) of sodium metaarsenite. 9 weeks	Exposure to iAs caused the dysregulation of mitochondrial processes.
Li et al., 2021 [25]	Male mice (C57BL/6J), age—7 to 8 weeks	0 or 20 mg/L (0 or 20 ppm) sodium arsenite. 12 months	iAs exposure induced systemic and hepatic insulin resistance and decreased liver GLUT4 concentrations.
Liu et al., 2021 [26]	Mice (C57BL/6J), age—8 to 10 weeks	Drinking water or drinking water + 25 ppm sodium arsenite. 20 weeks	NRF2 and p62 are associated with iAs-mediated insulin resistance
Xenakis et al., 2022 [31]	Diversity Outbred male mice (J:DO JAX stock number 009376) generation 35, age—26 to 32 days	100 ppb iAs in drinking water for 26 weeks	Associations between iAs consumption and fasting blood glucose, plasma insulin, β-cell function, and insulin resistance manifested as significant interactions between iAs and body weight/composition.

DM: Diabetes mellitus; DM2: Diabetes mellitus type 2; As: Arsenic; iAs: Inorganic arsenic; IL-1β: Interleukin 1 beta; TNF-α: Tumor necrosis factor alpha; PPARγ: Gamma-type peroxisome; ppm: Parts per million; SIRT3: Sirtuin 3; HOMA-IR: Insulin resistance index.

## Data Availability

Not applicable.
